# Cluster analysis for DNA methylation profiles having a detection threshold

**DOI:** 10.1186/1471-2105-7-361

**Published:** 2006-07-26

**Authors:** Paul Marjoram, Jing Chang, Peter W Laird, Kimberly D Siegmund

**Affiliations:** 1 Department of Preventive Medicine, Keck School of Medicine, University of Southern California, Los Angeles, CA 90089, USA; 2 Norris Cancer Center and Departments of Surgery and Biochemistry & Molecular Biology, Keck School of Medicine, University of Southern California, Los Angeles, CA 90089, USA

## Abstract

**Background:**

DNA methylation, a molecular feature used to investigate tumor heterogeneity, can be measured on many genomic regions using the MethyLight technology. Due to the combination of the underlying biology of DNA methylation and the MethyLight technology, the measurements, while being generated on a continuous scale, have a large number of 0 values. This suggests that conventional clustering methodology may not perform well on this data.

**Results:**

We compare performance of existing methodology (such as k-means) with two novel methods that explicitly allow for the preponderance of values at 0. We also consider how the ability to successfully cluster such data depends upon the number of informative genes for which methylation is measured and the correlation structure of the methylation values for those genes. We show that when data is collected for a sufficient number of genes, our models do improve clustering performance compared to methods, such as k-means, that do not explicitly respect the supposed biological realities of the situation.

**Conclusion:**

The performance of analysis methods depends upon how well the assumptions of those methods reflect the properties of the data being analyzed. Differing technologies will lead to data with differing properties, and should therefore be analyzed differently. Consequently, it is prudent to give thought to what the properties of the data are likely to be, and which analysis method might therefore be likely to best capture those properties.

## Background

With the invention of new high-throughput technologies, researchers are using molecular features to identify novel cancer subtypes. Currently, the most commonly analyzed molecular feature is gene expression. In such experiments, expression values are measured for a large number of genes (1,000's) across a smaller number of samples (10's-100's). More recent studies have used high-throughput arrays to measure protein abundances, single nucleotide polymorphisms (SNPs), or DNA methylation [1-3]. SNPs and DNA methylation are a more stable characteristic than gene expression, since they are based on DNA, which has less biological temporal variation and greater analyte stability than RNA. We investigate the use of DNA methylation for the classification of samples into disease subtypes. Previous studies of colon and lung cancer have shown some success [4,5].

**Figure 1:**
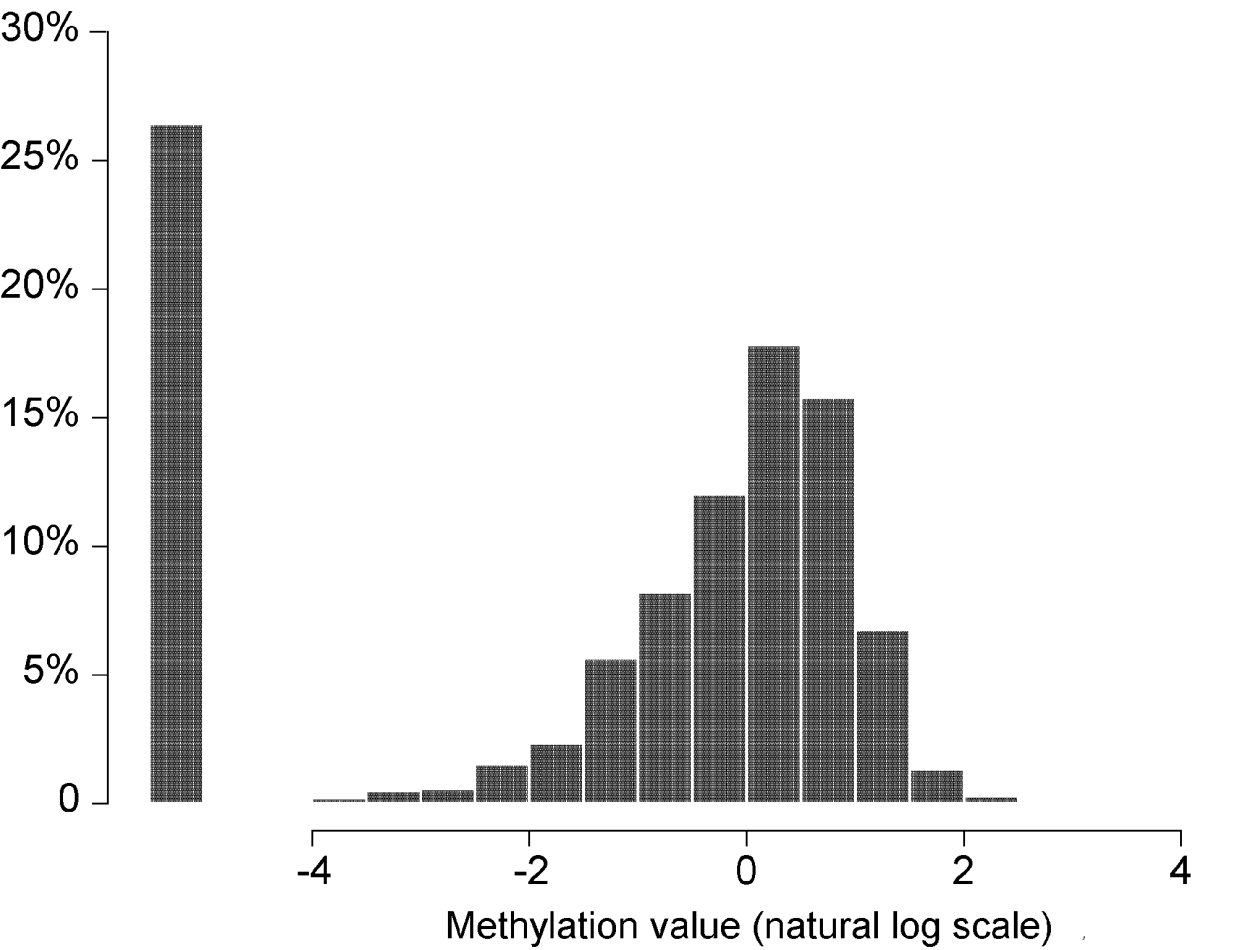
**Distribution of methylation values for 91 genes in 48 samples**. A histogram of methylation values (PMR) is shown. PMR values were transformed using the natural log. Zeros were assigned a value of -5.5, a value slightly below the lowest log-transformed value. The x-axis shows the methylation value. The y-axis shows the percentage of values in that range.

Currently there is no single platform for studying DNA methylation that is amenable to all study designs. As a result, measurements are obtained on some technology-dependent scale. In the data sets presented in this paper, DNA methylation is measured using the MethyLight technology [6]. Put briefly, this technology determines quantitative values from a standard curve of defined dilutions of a reference sample plotted (after taking logs) against the C(t) value (which is the cycle number at which the fluorescence signal crosses a detection threshold). The quantitative value for a sample is then derived via a linear regression on this curve. This value is normalized using a methylation-independent control reaction by taking the ratio. The ratio (multiplied by 100) of the normalized value for an experimental sample compared to that of a methylated reference sample represents the percent of methylated reference (PMR). The methylation-independent control reaction is used to normalize sample-to-sample variation in DNA quantity and integrity, while the methylated reference sample is used to control for the different efficiencies of reactions based on different oligonucleotide sequences. MethyLight probes are designed to detect a fully methylated sequence covering 5–10 CpG sites. Because of this stringent detection criterion, in some samples we do not detect any fully methylated molecules. This results in a distribution of PMR values that is quantitative and non-negative, but has an excess of zeros. We give an example of this in Figure 1 in which we plot the distribution of methylation values measured across a data set of 48 samples (see below for full details). One can clearly see the excess of zeros. Thus, the nature of our DNA methylation measurement is somewhat different than what is typical in a gene expression context, in which expression is conventionally reported on a scale corresponding to the real line (i.e., (log) expression can take any value, positive or negative). In previous work we have modeled this using a two-part model consisting of a Bernoulli distribution for the number of samples without detectable methylation and a log-normal distribution for the positively methylated samples [7]. Using simulations, we found that the Bernoulli-lognormal mixture can lead to lower classification error rates in the presence of zeros than a standard log-normal distribution.

It is conceivable that the two-part distribution is too flexible, resulting in a lack of efficiency due to over-fitting. Intuitively speaking, over-fitting is the phenomenon in which, once one has introduced sufficient parameters (i.e., genes) into the model to explain any signal present, any further parameters will merely introduce greater variability in the overall parameter estimates. This will lead to poorer performance in the final model. When using the MethyLight technology, it is likely that the unmethylated samples are due in part to a threshold of detection. This detection threshold is thought to be determined in part by sample-specific issues, such as template DNA quantity, but also by reaction-specific characteristics, such as the absolute and relative sensitivity of each reaction [8]. To capture the latter feature we propose to model a single detection threshold for each CpG region, reducing the total number of parameters in the model and hopefully resulting in a more successful fit. We propose to compare these new models to other analysis methods, such as k-means clustering. It is possible that the parsimonious nature of k-means clustering, for example, while not accurately modeling all features of the data, might more efficiently capture the key features. We explore this issue here.

We hypothesize that the fewer parameters required by the threshold detection model may translate into a lower classification error than the Bernoulli-lognormal model. These two methods, along with other clustering approaches, are compared on two data sets, one featuring lung cancer and the other featuring colon cancer. We then conduct a simulation study to evaluate the performance of our proposed methods and k-means clustering when the numbers of loci varies or when the studied genes show correlation within disease subtype.

### Data sets

*Lung cancer study *Virmani et al. studied DNA methylation of 24 CpG regions in a sample of 87 cell lines [5]. Out of the 24 regions, three had no detectable level of DNA methylation. Out of the 21 regions showing variable DNA methylation levels, seven were identified that could distinguish small cell from non-small cell lung cancer. We use these seven loci to cluster samples, comparing our results from the different cluster analysis approaches with the independently assigned histology.

*Colorectal cancer study *In a study at the University of Southern California, DNA methylation was measured on 91 genes for 48 colorectal cancer tissues [9]. These genes were pre-selected for showing differential methylation in tumor and adjacent normal tissue. Using a variety of different clustering routines, earlier analyses have identified 2–3 distinct clusters from these data, with the strongest evidence for two clusters, referred to as CIMP/no-CIMP (unpublished data). This classification of samples was confirmed by analysis of an independent data set [9]. We compare the results from the clustering approaches presented here to the classification of samples resulting from that previous work.

## Results

We analyze both real and simulated data sets using a variety of standard techniques, such as k-means clustering [10], using the software S-PLUS version 6.1 [11]. In addition, we employ two particular alternate models that are designed to capture the feature that there is a threshold below which methylation cannot be detected: Model 1, a Bernoulli-lognormal model; Model 2, a single threshold model. In Model 1, the threshold of detection varies across both CpG regions and disease subtype; in Model 2 the threshold varies by CpG region but is constant across disease subtypes (thereby allowing the use of fewer parameters). Further details of these two models are given in the Methods section.

### Real data

**Figure 2:**
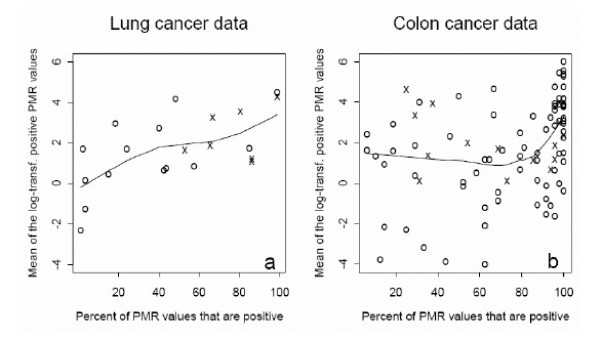
**Mean of the log-transformed positive PMR**. Mean of the log-transformed positive PMR values are plotted against the percent of positive PMR values in (a) lung cancer cell lines (87 samples/21 genes; x: small cell-predicting genes/o: non predicting genes) and (b) colon cancer tissue (48 samples/91 genes; x:CIMP-predicting genes/o: non predicting genes).

Figure 2 shows a plot of the mean of the log-transformed positive PMR values against the proportion of PMR values for the lung and colon cancer data sets. The curve is produced by the lowess() function in SPLUS, which fits a smooth, robust, locally linear curve to represent trend in a scatterplot [12]. A positive correlation between these two measures supports the idea of a threshold for detecting positive DNA methylation. A few zeros suggest that the mode of the distribution of measurements (e.g. top of the bell-shaped curve) lies far to the right of the threshold of detection allowing for high estimates of the mean value (high proportion positive/high mean). An abundance of zeros suggest that the mode of the distribution is below the minimum level of detection so that the mean of the positive values would be low (low proportion positive/low mean). We see a strong positive association across the entire range of measurements for the lung cancer samples. The association is only seen among the genes with a high proportion of methylated samples for the colon samples. Comparing the stability of the estimates from the two data sets, the estimates from the lung cancer data should be more stable due to the larger number of samples measured (n = 87 vs n = 48). The lung cancer data, with its associated greater stability, is supportive of the biological intuition that lies behind the specification of the second of our alternative models. The colon cancer data are clearly less supportive of this model, and this might lead the model to perform less well on that data. (However, we note that the greater number of genes in that data set will have the effect of making clustering easier, which acts to improve predictive power.)

In Table 1 we show the performance of our two models and a variety of popular alternative clustering methods on both the colon and lung cancer data. Specifically, we also include results for k-means clustering, Partitioning Around Medoids (PAM) [13], divisive hierarchical cluster analysis (HCD), the MCLUST software of [14], and self-organizing maps (SOM) [15]. K-means and divisive hierarchical clustering was performed using Euclidean distance. The self-organizing map analysis was undertaken using the som() function in R. We pre-determine the number of clusters at two, and then assess performance by calculating the "misclassification rate", which is the proportion of data that are clustered into the incorrect group. We also show two measures of cluster integrity that are independent of correspondence to any 'phenotype': silhouette width and Dunn's index. (See [16] and [17] for detailed definitions.) Larger values of these statistics correspond to better-clustered data, while smaller values correspond to data for which the clustering is less-well defined.

We see that there is a substantial difference in performance across the two data sets. This is a consequence of the difference between the number of genes in each data set. The colon data contains methylation values for 91 genes on 48 samples. In contrast, the lung data contains measurements for only 7 genes, albeit for a larger number of samples. The results suggest, perhaps unsurprisingly, that for the variation in methylation level across these samples, 7 genes are not enough to accurately classify the data (although it is interesting to note that our methods are among the best on that data, even though the signal is poor). Below, we go on to investigate the issue of how many genes are required to successfully cluster the data.

A second point is that, with the exception of self-organizing maps, there is relatively little difference between the analysis methods applied to the colon data. The single threshold model does appear slightly more effective, as per the intuition outlined earlier, but the difference is not substantial. Curiously, self-organizing maps appear to perform poorly here, although we note with interest that, in a microarray context, [18] also found self-organizing maps to perform poorly on lung and colorectal cancer data whereas they obtained nearly perfect results when applying the same methodology to a variety of other cancer types. We also tried an agglomerative hierarchical clustering analysis, but this performed very poorly. A general point here is that divisive clustering is likely to perform better than agglomerative clustering when looking for a few large clusters (since it is a top-down method, rather than the bottom-up approach of the agglomerative method) [13]. There also appears to be a poor correspondence between independent measures of cluster integrity and the degree to which those clusters correspond to sample type. This indicates that there is a difference between tightness of clustering and correspondence of those clusters to external clinical criteria.

### Simulation study

We now undertake a simulation study, based on the colon data (see Methods for details), in which we investigate the relative accuracy of the models as the number of genes decreases and as the correlation among genes in the same family increases. Since k-means performs relatively well on our data, we use it as a representative of the alternative methods considered in Table 1 and therefore include results for k-means with those for our methods in the simulation study. Our aim is to explore whether it is generally true that k-means and our proposed models have similar performance, and, perhaps more importantly, to assess the dependency of accuracy of classification upon the number of genes for which methylation is measured.

In Table 2 we present results showing how the misclassification rate depends on the analysis model used, as well as the percentage of genes for which methylation is measured. Simulated genes are classified as "CIMP-predicting" and "Other" (see Methods) according to how well the simulated values correspond to CIMP status. In order to reduce unnecessary noise, if *P *percent of genes were sampled we ensured that *P *percent of each of the "CIMP-predicting" and "Other" classes of genes was sampled. In the bottom row of Table 2 we show how accurate the classification is if just the CIMP-predicting genes are measured. We report the mean misclassification rate over 50 simulated data sets for each scenario, along with the standard error of the mean.

In all cases we see that the single threshold model out-performs the other two approaches. This illustrates our intuition that the model that respects the form of our measurements, while keeping the number of parameters to a minimum, will perform best. The Bernoulli-lognormal and k-means approaches are similar for the data sets having fewer numbers of genes. As the number of genes grows beyond 70, the k-means clustering performs better. This illustrates the issue of over-fitting we discussed earlier. Even though k-means mis-specifies the distribution of our data measurements, the fact that the model requires fewer parameters results in a more efficient fit, with a lower misclassification rate. We also note that in order to classify the samples with a high degree of accuracy it is sufficient to have a set of 36 or so genes. However, in such a scenario we still perform more poorly than a situation in which we have the 15 most informative (i.e., CIMP) genes.

In the above scenario, we simulated data where the correlation among genes was completely explained by the cluster to which they belonged, and, within cluster, the genes were completely independent. In reality it is likely that certain subsets of genes have correlated methylation values within cluster as well, so we now explore how such additional correlation might affect the performance of our analysis methods. Table 3 presents results for these models showing how the misclassification rate varies depending on the within-group correlation for the two gene clusters. The primary effect to note is that the ability to successfully cluster the data is a decreasing function of the correlation present within that data. This is intuitively sensible. Two correlated genes carry less information than two independent genes since the information in the first gene can be used to predict the information in the second gene. As the correlation between genes increases, the amount of extra information imparted by the other genes decreases. Thus performance degrades as correlation increases. It is also interesting to note that the single threshold model now performs similarly to the Bernoulli-lognormal model. It seems that the advantage originally seen by the single threshold model disappears when the correlation among genes within gene cluster is mis-specified. This time k-means clustering performs worse than the Bernoulli-lognormal model. The model-misspecification in the k-means model (with fewer parameters) is greater, resulting in the highest misclassification rate.

## Discussion

In this paper we have extended current mixture models for cluster analysis to include a detection threshold for data having an excess of zeros. Our motivation for doing this was to better reflect the underlying biological properties of the data and measurement experiment. By doing so we hoped to improve the performance of our analyses. Analyses such as those we present here might have several biologically significant aims. Firstly, while one might know, or suspect, that a given set of genes might be related to a specific cancer type, it does not necessarily follow that those genes carry enough information to provide a reliable diagnostic/discriminant function. Our analysis makes it clear that this is not true for the genes we study in the case of lung cancer for example. Secondly, one might use an analysis such as this to determine the importance of particular genes when differentiating between tissue types. For example, one might conduct analyses in which the gene is included/excluded and compare performance.

We tested our methods by application to DNA methylation data from two studies, one of lung cancer and another of colorectal cancer. Results from one data set, for lung cancer, were uniformly poor due to the low number of genes for which methylation was measured. However, on the other data we saw that extending our models does indeed improve clustering performance compared to methods, such as k-means, that do not explicitly respect the supposed biological realities of the situation (and, thereby, the likely properties of the data).

There is a general point to be made here. We, and others, have demonstrated that methylation can be used to categorize data. However, in this paper we have also shown that the performance of any given analysis method is likely to depend upon how well the assumptions of that method reflect the properties of the data being analyzed. Differing technologies will lead to data with differing properties, and should therefore be analyzed differently. This will likely be true of alternative platforms for measuring the same biological property (e.g. methylation), as well as for platforms that measure other features (e.g. expression arrays). Given this, it is prudent to give some thought to what the properties of the data are likely to be, and to which analysis method might therefore be able to best capture those properties. In this paper we have demonstrated that the method we introduce here, which specifically respects the mixed-model feature of the data, performs better than existing methods on data with that same property. We do not claim that our method will perform well on all data sets, regardless of their likely features, but rather we stress that most power is gained by choosing a method that captures the key properties of the data. Unfortunately, it is impossible to give generalities here, but we hope to have demonstrated that some thought is necessary before applying any particular analysis tool to any (or all!) given data.

## Methods

We assume that the methylation data, *Y*_*gs*_, for gene *g *on sample *s *has the following characteristics. With some probability *p*_*gs *_we have *Y*_*gs *_*= 0*; otherwise, *Y*_*gs *_follows some continuous distribution function *F*_*gs*_, with mean *μ*_*gs *_and variance σ^2^. In its full generality, such a model allows for different values of *μ*_*gs*_, *p*_*gs *_and *F*_*gs *_for all samples and genes. We assume we have data measured for *N*_*G *_genes on *N*_*S *_samples. For each method we propose below we assume that for each cluster the mean methylation value *μ*_*gs *_is constant for each gene. So, for cluster *c*, we assume *μ*_*gs *_= *μ*_*g*_*(c) *for all subjects *s *in *c*.

### Model 1. Bernoulli-lognormal model

In this model we assume that, for each gene, in each sample, there is a probability *p*_*gs *_that the experiment returns a methylation value of zero (i.e., *Y*_*gs *_= *0*). Otherwise, (with probability *1-p*_*gs*_) the gene returns a value drawn from a continuous distribution *F*_*gs *_(assumed to be normal on a log-scale) with mean *μ*_*gs *_and variance assumed to be 1 (after an appropriate re-scaling). We assume that *p*_*gs *_is constant for each given gene within each cluster of samples. i.e., in cluster *c *(say), we have *p*_*gs *_= *p*_*g *_*(c) *for all *s *in *c *(i.e., the zero probability can vary for each gene and for each cluster, but, for a given gene, is constant for all samples within a given cluster). For a two cluster model, the number of parameters in this setting is *4N*_*G *_(since *p*_*gs *_varies across clusters).

### Model 2. Single threshold model

In this model we assume that genes return a methylation of zero because their true methylation value falls below a given detection threshold. Thus, we assume that *Y*_*gs *_is a function of a true, unobserved methylation value *Z*_*gs*_, and that, on a log-scale, *Y*_*gs *_= *Z*_*gs *_if *Z*_*gs *_≥ *τ*_*gs*_, and *Y*_*gs *_= *ln(0) *= -∞ otherwise. Thus, *p*_*gs *_is parameterized in terms of a value *τ*_*gs *_which corresponds to a threshold below which methylation cannot be detected. Since we believe that the truncation point depends largely upon the biochemical properties of a given probe, we set *τ*_*gs *_= *τ*_*g *_for all *s *(i.e., there is a single detection threshold for each gene and this threshold is constant across clusters). Using the notation from model 1, it follows that *p*_*gs *_= *p*_*g *_for all *s*. The zero probability still varies for each gene but does not vary with sample cluster, in contrast to model 1. In a two cluster context, the number of parameters in this model is 3*N*_*G*_, rather than the *4N*_*G *_parameters in model 1.

### Estimation

We employ a Markov chain Monte Carlo clustering algorithm, analogous to k-means clustering, to fit the models given above. We implement a Metropolis-Hastings algorithm [19,20] which results in a posterior distribution for the cluster allocation of the samples (and the related parameter space), rather than the single 'best' clustering that results from most cluster analysis methods. We cluster samples into 2 groups in an attempt to differentiate 'normal' from 'abnormal' DNA methylation profiles.

At any given iteration of our algorithm, samples are allocated into one of two clusters. Each cluster, *c*, corresponds to a vector of values *(μ*_1_*(c)*,...,*μ*_*G*_*(c);p*_1_*(c)*,...,*p*_*G*_*(c)) *which determines the mean methylation value and zero probability at each gene for samples within that cluster. We proceed in a manner analogous to the popular k-means algorithm. For model 1, we set *μ*_*g*_*(c) *equal to the mean of all non-zero methylation values for gene *g *for samples within that cluster, while *p*_*g*_*(c) *is set equal to the proportion of samples with non-zero methylation values for gene *c *in that cluster. In model 2 we set the truncation value *τ*_*g*_*(c)*, which determines *p*_*g*_*(c)*, equal to the smallest methylation value observed at gene *g *for all samples (regardless of whether they are in that particular cluster); We define *μ*_*g*_*(c) *= *Σ*_*s*_*max(τ*_*g*_*(c),Y*_*gs*_*)/N*_*c*_, where *N*_*c *_is the number of samples currently assigned to cluster *c *(i.e., *μ*_*g*_*(c) *is defined to be the mean of the methylation values for that gene for all samples in the cluster, treating zero values as if they were equal to the value *τ*_*g*_*(c) *at which the distribution is truncated). As such, the fitted value of *μ*_*g*_*(c) *is not equal to the maximum likelihood estimate (MLE) since truncated values are in fact less than or equal to *τ*_*g*_*(c)*. However, we felt that the extra computational burden required to calculate the true MLE would not result in a measurable improvement in performance. Between iterations, changes are proposed to the way in which samples are clustered. In particular, a single sample is chosen to be moved to the other cluster. The new state is then "accepted" with a probability determined by the Hastings Ratio [19,20] in which case it becomes the current state. Otherwise the newly proposed state is rejected and the process returns to its previous state. After a suitable burn-in period (10000 iterations) we begin to output the sample clustering at each iteration of the algorithm, and a misclassification rate calculated from that clustering. We report the mean misclassification rate over the next 10000 iterations of the algorithm. Formally, our misclassification rate is calculated as follows. Assume the (unobserved) truth is that our data falls into two groups: *A *and *B*. At any given iteration, our analysis will cluster the data into two clusters, C and D. We calculate the number of samples *n*_*A *_that are misclassified if group A corresponds to cluster C, while group B corresponds to cluster D. We also calculate the number of samples *n*_*B *_that are misclassified if group A corresponds to cluster D, etc. We report the misclassification rate for that iteration as the minimum of *n*_*A *_and *n*_*B *_divided by the total number of samples.

Our approach is closely related to that of k-means clustering, the results of which we also present, but differs in two respects. Firstly, we explicitly allow for data in which there is a probability mass at zero. Secondly, we obtain a posterior distribution for all possible clusterings rather than the single 'best' cluster that results from a k-means algorithm. By doing so we better allow for the uncertainty due to the unknown true sample clustering.

### Simulation study

We explore the question of when the threshold mixture-model will provide a lower classification error than the Bernoulli-lognormal mixture and k-means models. In order to do this we simulate data analogous to the colorectal cancer study discussed above. (All models perform poorly on data simulated to mimic the lung cancer study due to the low predictive ability of the small number of genes for which methylation was measured [unpublished results, but see Table 2].) In doing so, we model the biological intuition that the preponderance of methylation values at zero is likely to be the result of low 'true' methylation values that are measured as zeros due to the threshold detection of the corresponding probe. Thus we simulate data according to a threshold model in which, on a log-scale, genes have an unobserved 'true' methylation, *m*, that is distributed according to a normal distribution with a given mean and variance. The methylation value, *M*, that is recorded is equal to *m *if *m *is greater than a gene-specific threshold value, and is equal to zero otherwise. In order to make the simulation study agree closely with the observed colorectal data, we set the mean, standard deviation and threshold value of the methylation distribution for a given gene in such a way that we maintain summary properties of that data. Toyota et al. [4] proposed that a subset of colorectal cancers having a high frequency of DNA methylation could be identified by a subset of genes that were methylated in cancer but not normal tissue. The subset of cancers is said to have the CpG Island Methylator Phenotype (CIMP). In our data there are 15 of these "CIMP-predicting" genes.

We calculated statistics for the percentage of samples for which genes had a non-zero methylation, and the mean of the (non-zero) methylation values for that gene within two classes, depending on whether the gene was "CIMP-predicting" or "Other". Statistics were also broken down by "CIMP"/"non-CIMP" tumor status. The observed summary statistic values are shown in Table 4. Interestingly, even the genes in the "Other" group seem to show a different average DNA methylation level between CIMP and non-CIMP samples. This suggests that the association of the genes with CIMP status of the tumor actually lays on a continuum and is not as simplistic as our grouping into two sets. Nonetheless, for simplicity we define our simulation study using these two gene classes.

In order to match these data as nearly as possible, we simulated two classes of genes, corresponding to the "CIMP-predicting" and "Other" classes. Within each class, genes were identically distributed from a *N(μ,σ*^2^*) *distribution, where *μ *was chosen to match the values given in Table 4; *σ*^2 ^and the truncation value *p *were set in order to match the percent positive values in Table 4 as closely as possible. This led us to use *σ *= *2.75 *and *τ *= *0.2 *for all genes. The resulting percent positive values for the CIMP-predicting genes were 92% and 60% for groups one and two respectively, whereas for the "Other" genes the figures were 84% and 71%. Thus we feel our simulated data is representative of the observed colorectal data.

## Authors' contributions

PM, JC and KDS conducted the statistical analysis and model development. The molecular data were produced in PWL's lab. PWL and KDS conceived the study. PM and KDS prepared the manuscript. All authors read and approved the final manuscript.

## Figures and Tables

**Table 1:** Misclassification rates for analysis of two observed data sets

	Colon cancer Error rate (Silhouette width/Dunn's index)	Lung cancer Error rate (Silhouette width/Dunn's index)
Bernoulli-lognormal model	0.04 (0.15/1.16)	0.31 (0.29/1.19)
Single threshold model	0.01 (0.17/1.20)	0.30 (0.29/1.25)
k-means	0.04 (0.17/1.18)	0.38 (0.35/1.25)
PAM	0.04 (0.17/1.18)	0.30 (0.29/1.18)
HCD	0.04 (0.15/1.18)	0.38 (0.35/1.25)
MCLUST	0.04 (0.14/1.12)	0.36 (0.30/1.24)
SOM	0.21 (0.12/1.14)	0.37 (0.34/1.20)

**Table 2:** Misclassification rates (standard error) by the number of genes selected (48 samples)

			Misclassification rate (SE)		
Percentage of genes selected	No. of CIMP genes	No. of non-CIMP genes	Bernoulli- lognormal	Single threshold	k-means

100%	15	76	0.018 (0.020)	0.006 (0.009)	0.010 (0.002)
80%	12	61	0.030 (0.004)	0.009 (0.002)	0.020 (0.004)
60%	9	46	0.067 (0.007)	0.022 (0.003)	0.070 (0.013)
40%	6	30	0.147 (0.013)	0.073 (0.009)	0.140 (0.014)
20%	3	15	0.265 (0.012)	0.177 (0.013)	0.283 (0.016)
*CIMP-predicting genes only (N = 15)*			0.035 (0.004)	0.020 (0.004)	0.037 (0.005)

**Table 3:** Misclassification rates for different pair wise correlations within the two gene clusters (48 samples/91 genes)

	Misclassification rate (SE)		
Pair wise correlation	Bernoulli- lognormal	Single threshold	k-means

0.01	0.024 (0.004)	0.013 (0.004)	0.024 (0.004)
0.05	0.076 (0.008)	0.062 (0.006)	0.119 (0.012)
0.1	0.155 (0.012)	0.169 (0.015)	0.220 (0.015)
0.2	0.229 (0.013)	0.235 (0.012)	0.342 (0.012)

**Table 4:** Summary statistics for colorectal cancer data

	CIMP group (N = 11) Mean (%positive)	Non-CIMP group (N = 37) Mean (%positive)
CIMP-predicting genes (N = 15)	4.15 (92)	0.95 (61)
Other genes (N = 76)	3.02 (82)	1.74 (72)
